# Can drones count gulls? Minimal disturbance and semiautomated image processing with an unmanned aerial vehicle for colony‐nesting seabirds

**DOI:** 10.1002/ece3.4495

**Published:** 2018-12-04

**Authors:** Graham P. Rush, Lucy E. Clarke, Meg Stone, Matt J. Wood

**Affiliations:** ^1^ School of Natural and Social Sciences University of Gloucestershire Cheltenham UK; ^2^ Department of Environment and Geography University of York York UK

**Keywords:** aerial survey, image classification, *Laridae*, monitoring seabird, population ecology

## Abstract

Accurate counts of wild populations are essential to monitor change through time, but some techniques demand specialist surveyors and may result in unacceptable disturbance or inaccurate counts. Recent technological developments in unmanned aerial vehicles (UAVs) offer great potential for a range of survey and monitoring approaches. They literally offer a bird's‐eye view, but this increased power of observation presents the challenge of translating large amounts of imagery into accurate survey data. Seabirds, in particular, present the particular challenges of nesting in large, often inaccessible colonies that are difficult to view for ground observers, which are commonly susceptible to disturbance. We develop a protocol for carrying out UAV surveys of a breeding seabird colony (Lesser Black‐backed Gulls, *Larus fuscus*) and subsequent image processing to provide a semiautomated classification for counting the number of birds. Behavioral analysis of the gull colonies demonstrated that minimal disturbance occurred during UAV survey flights at an altitude of 15 m above ground level, which provided high‐resolution imagery for analysis. A protocol of best practice was developed using the expertise from both a UAV perspective and that of a dedicated observer. A GIS‐based semiautomated classification process successfully counted the gulls, with a mean agreement of 98% and a correlation of 99% with manual counts of imagery. We also propose a method to differentiate between the different gull species captured by our survey. Our UAV survey and analysis approach provide accurate counts (when comparing manual vs. semi‐automated counts taken from the UAV imagery) of a wild seabird population with minimal disturbance, with the potential to expand this to include species differentiation. The continued development of analytical and survey tools whilst minimizing the disturbance to wild populations is both key to unlocking the future of the rapid advances in UAV technology for ecological survey.

## 1 INTRODUCTION

If the population dynamics of wild animal populations are to be understood and effective conservation management to take place, accurate estimates of population size are essential. However, some species are challenging to survey, inhabiting inaccessible locations that are difficult to visit or to observe (e.g., cliff‐nesting or colony‐nesting seabirds) or are susceptible to disturbance by fieldworkers or recreational activity (Giese, 1996; Kerlinger et al., 2013; Schlacher, Nielsen, & Weston, 2013). Unmanned aerial vehicles (UAVs) present an opportunity to overcome such challenges, by using this increasingly affordable technology to gather aerial views of animal populations. However, the rapid development of UAVs in wildlife research has brought its own challenges: how to conduct UAV surveys without undue disturbance to the local population, and how to handle and analyze large amounts of aerial imagery to ensure that UAVs develop into a useful tool in ecological studies.

### 1.1 Monitoring seabird populations

The United Kingdom hosts a relatively large number of seabirds compared to areas of similar latitudes because of the highly productive surrounding seas (Nager & O'Hanlon, 2016). The abundance and distribution of seabirds have been monitored since the late 1960s (Cramp, Bourne, & Sanders, 1974; Lloyd, Tasker, & Partridge, 1991; Mitchell, Newton, Ratcliffe, & Dunn, 2004), enabling the extraction and analysis of population trends which can promote understanding of the underlying factors behind them and be used to influence decision making and provide information about the marine environment in which they live (Furness & Greenwood, 1993). Seabirds require predator‐free breeding sites with access to open seas, which are often in large colonies in isolated locations such as oceanic islands or sea cliffs, which can make monitoring populations difficult. A range of monitoring protocols have been developed to manually survey different colony‐nesting seabird species, but the challenges of access, viewing, and disturbance remain, especially for gull species (*Larus* spp.; Walsh et al., 1995).

Substantial declines have been observed in coastal colonies of gulls, with some now listed as species of conservation concern (Eaton et al., 2013). Surveys of gull populations are traditionally carried out by trained surveyors using methods such as point counts, transects, or walk‐through surveys (Bibby, Burgess, Hill, & Mustoe, 2000). Point counts are made by an observer from a vantage point over a colony; however, inconsistencies arise due to nesting in dense vegetation, inaccessible subcolonies being disregarded, observer difficulties in the use of optical equipment in field conditions, and observer bias from different surveyors with varying levels of expertise (Bibby et al., 2000). Walk‐through surveys are utilized specifically to count both numbers of nests and eggs within colonies and produce accurate results that are important in determining breeding success and survival rates of chicks. However, they present logistical challenges, requiring a large number of people and can take considerable time, and can be challenging in determining which nest belongs to which gull species. Significant disturbance can be caused during human incursions into the colony, including increased levels of intraspecific aggression and/or predation of eggs and chicks, and increased nest abandonment (Carney & Sydeman, 1999). Disruption of the surrounding habitat may also be of concern, such as damage to vegetation and the nests of burrow‐nesting species in fragile habitats such as Atlantic Puffin, *Fractercula arctica* and Manx Shearwaters, *Puffinus puffinus* (pers. obs. by authors).

### 1.2 Remote monitoring of seabird populations

Aerial images have the advantage of a permanent record that can be viewed any number of times, enabling studies at temporal and spatial scales not feasible by traditional visual count methods (Lillesand, Kiefer, & Chipman, 2015). Recent technological developments have increased the availability of aerial imagery and the potential for remote sensing applications in ecological studies, using either satellite‐derived images or unmanned aircraft (Anderson & Gaston, 2013) to capture imagery. For example, high‐resolution satellite‐borne imagery has been utilized to locate and count breeding colonies of larger seabirds such as Emperor Penguins, *Aptenodytes forsteri* (Fretwell, Trathan, Wienecke, & Kooyman, 2014; Fretwell et al., 2012), and Wandering Albatrosses, *Diomedea exulans* (Fretwell, Scofield, & Phillips, 2017). However, the high cost of such imagery, the potential for cloud to obscure the area of interest when satellite‐derived images are available, and a lack of control over the acquired resolution preclude its use in many cases (Loarie, Joppa, & Pimm, 2007). The development of UAVs, commonly referred to as drones or remotely piloted aircraft (RPA), offers the opportunity to bypass some of these difficulties whilst allowing the user greater control over the collection of aerial imagery at a suitable scale and resolution (Lillesand et al., 2015), thus permitting accurate counts. UAVs are small (typically <7 kg in weight), powered aerial vehicles that come in a variety of platforms including fixed‐ and rotary‐winged aircraft, kites, and balloons (Woodget, Carbonneau, Visser, & Maddock, 2014) that can carry a payload (i.e., a camera) and are able to be flown remotely or autonomously.

### 1.3 Unmanned aerial vehicles as a solution

Following initial exploration of the utility of UAVs in wildlife monitoring (Hodgson & Koh, 2016; Jones, Pearlstine, & Percival, 2006; Mulero‐Pazmany et al., 2017), a number of seabird colonies have been counted using this approach. This has typically involved the collection of images by UAV survey followed by manual image counting of the number of individuals, for example Black‐headed Gulls, *Chroicocephalus ridibundus* (Sardà‐Palomera et al., 2012) and Common Terns, *Sterna hirundo* (Chabot, Craik, & Bird, 2015), with 93%–96% accuracy compared to ground counts. Hodgson, Baylis, Mott, Herrod, and Clarke (2016) used similar manual methods to count breeding individuals in colonies of three seabird species and, importantly, demonstrated an increased precision of population counts derived from UAVs compared to those from ground‐based observer counts.

Whilst the use of UAVs presents the enticing opportunity for more accurate population counts, the volumes of data become unwieldy for larger populations and may thus be subject to observer error during manual counts of individuals from colonywide imagery. These considerations drive our use of image classification to automate counts of individuals and thereby unlock the potential for wider scale adoption of UAV survey approaches. Grenzdörffer (2013) combined the merging of UAV‐derived images with the automatic counting of Common Gulls, *Larus canus*, using an approach similar to Fretwell et al.'s (2012, 2014) detection of image spectral signatures, indicating that individual birds could be automatically identified against a distinct background with reasonable accuracy (97.6%).

The bird's‐eye view offered by UAV surveys can only be useful if the imagery can be captured at sufficient resolution to be useful for population counts. High‐resolution cameras of small size and weight are now available, but it is necessary for a UAV to fly low enough to capture useful images without causing unacceptable disturbance on ethical grounds or that could reduce the clarity of the images that are captured. This consideration seems to have been overlooked in early studies of wild animal monitoring, but has received much attention more recently.

Grenzdörffer (2013) does briefly remark on the optimal flying distance and the response of birds, but this was not quantified. Vas, Lescroël, Duriez, Boguszewski, and Grémillet (2015) reported little effect of UAV color, flight speed, and angle of approach toward nonbreeding groups of semicaptive Mallards, *Anas platyrhynchos* and Greater Flamingos, *Phoenicopterus roseus* and wild Common Greenshanks, *Tringa nebularia*, flying as close as 4 m without noticeable response. However, as the birds in the survey were semicaptive this could have influenced the results as they are more adjusted to human disturbance, and the authors acknowledge the flying height may be different depending on the species and the breeding status. Weimerskirch, Prudor, and Schull (2017) explored the impact of flying height on 11 seabird species on the Crozet Islands, Southern Indian Ocean. They found that at 50 m, there was minimal disturbance with only one species showing a reaction, whereas at 10 m, all species demonstrated behavioral stresses, but again, the response was species‐dependent with some showing little behavioral response when flying at <5 m. Brisson‐Curadeau et al. (2017) assessed the impact of using a UAV on cliff‐nesting Arctic seabirds and confirmed that the response was species‐dependent and suggested baseline tests to determine the species‐specific responses, and encouraged habituation flights before capturing data from 20 m above the ground. McEvoy et al. (2016) determined that 40 m was a suitable height for flying a small UAV over nonbreeding wildfowl, with disturbance noted at flying heights below this, or whilst the UAV rapidly changed direction or altitude when above the birds. Hodgson et al. (2018) carried out UAV surveys on life‐size replica colonies and found no significant increase in count accuracy was achieved by obtaining imagery from heights lower than or equal to 90 m, but this needs to be verified using real colonies that have complex vegetation and background patterns to extract data from. The small but rapidly developing body of research conducted thus far suggests that with prudent flying, UAV‐based ornithological research has a multitude of possibilities which is largely dependent on species size and how distinctive they are from the surrounding habitat, but no unified protocols for the ethical use of UAV currently exist.

This work therefore aims to contribute to the development and application of UAV for avian surveys, addressing three research questions:

1. What is the best practice for flying a UAV above breeding gull colonies?
2. Can individuals within a Lesser Black‐backed Gull colony be identified and be counted using a semiautomated system?
3. Can a semiautomated identification system recognize individuals of different gull species?

## 2 MATERIALS AND METHODS

### 2.1 Study site

**Figure 1:**
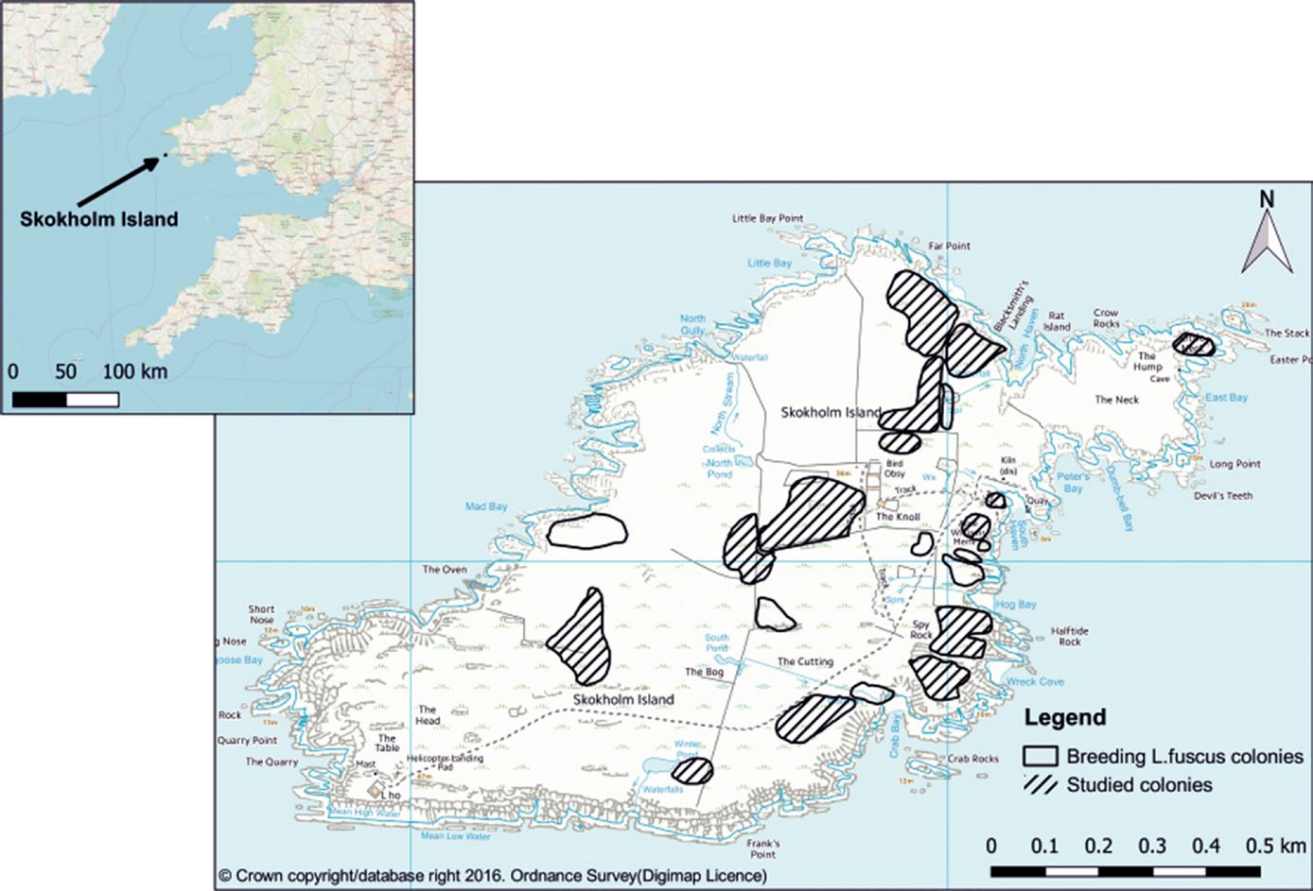
Location map of Skokholm Island and position of *Larus fuscus* breeding colonies, indicating those that were used in this study

Field data collection was carried out on Skokholm Island, Wales, UK (Latitude: 51.69, Longitude: −5.28, Figure 1) between May 16, 2016, and May 23, 2016. Surveying was timed to coincide with island gull counts, which are carried out during the incubation period when birds are most closely associated with their nest. Skokholm Island is of national and international importance for its seabird populations (designation of Special Protected Area; Thompson, 2007). The flora of the Island is mainly submaritime, including grassland, boggy areas, and coastal vegetation. The island population in 2016 of approximately 1,400 breeding pairs of Lesser Black‐backed Gulls is divided into 22 subcolonies (Figure 1, Brown & Eagle, 2016); these subcolonies contain primarily Lesser Black‐backed Gulls rather than being mixed species. The population decline on the island (Eaton et al., 2013) has been linked with low breeding success, likely due to a reduction in food availability during the rearing period (Thompson, 2007).

### 2.2 Unmanned aerial vehicle survey

A DJI Inspire 1 quadcopter UAV was used for all aerial flights. This was fitted with a 12 megapixel DJI FC350 camera, with a rectilinear, curved lens designed to eliminate distortion, and a 20 mm focal length allowing for wide angle pictures with minimal fish‐eye with a resolution of 72 DPI. This UAV proved able to fly and remain stable in wind speeds of up to ≈15 mph.

The camera used had an internal global positioning system (GPS) that enabled georeferencing (i.e., positioning in the correct physical location) of each image. However, ground control points (GCPs) are required in most photogrammetry activities (Lillesand et al., 2015) and are of central importance in successful orthorectification projects by significantly increasing the accuracy of the final product (Liu, Zhang, Peterson, & Chandra, 2007). The process of orthorectification used here is the process of removing the effects of image perspective and relief to create an image with a constant scale and features in their true relative positions (i.e., an orthorectified image). Therefore, a Leica differential global positioning system (DGPS) GS16 was used to collect 170 GCPs from around the Island. Identifiable points were selected that could be accessed without causing disturbance to the birds, were easily visible on the collected imagery, and represented variations in elevation across the island.

### 2.3 Unmanned aerial vehicle flight protocol

**Figure 2:**
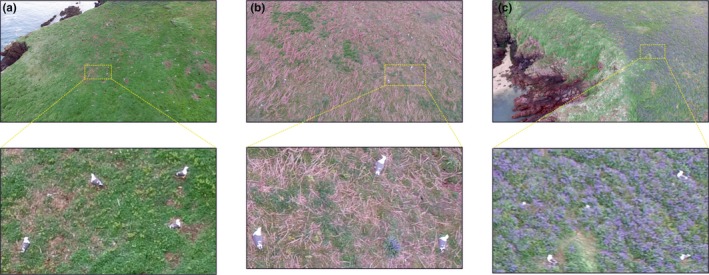
Example imagery captured by the unmanned aerial vehicle flown at 15 m altitude with zoomed areas showing the spatial resolution achieved and *Larus fuscus* identification on (a) open grassland, (b) rough grass/bracken, *Pteridium* spp. scrub, and (c) bluebells, *Hyacinthoides nonscripta*

For each UAV survey, a take‐off and landing site was chosen that was accessible from the footpath network (to avoid damage to the fragile habitats on Skokholm) and an adequate distance from the colony so as not to disturb the birds as recommended by Vas et al. (2015), in this case a minimum of 50 m. Survey altitude was determined by a test flight at one subcolony, under dual expert observation by one of the Island wardens and a trained seabird ecologist, both familiar with gull behavioral responses and able to assess any behavioral disturbance. At 40 m above the surface of the ellipsoid (m.a.s.e.)—that is, the elevation above the starting position of the UAV—gulls appeared to notice the presence of the UAV and were alert but not disturbed. Initially, the UAV was lowered from a stationary position at 40 m.a.s.e. directly above the subcolony, which caused immediate and widespread alarm in the subcolony (flights and alarm calling), similar to the predator response noted by Brisson‐Curadeau et al. (2017); therefore, this practice was immediately discontinued. Thereafter, the UAV was flown at a steady speed (3–4 ms^−1^) whilst above the subcolony. The UAV was then flown approximately 20 m to the side of the subcolony, and the height of the UAV was lowered by 5 m as smooth a flight as conditions allowed, and survey flight resumed over the subcolony. No observed disturbance was caused down to 15 m.a.s.e; at this elevation, a few birds took to the air, but no incubating birds took flight and no aggressive interactions were observed. Below 15 m.a.s.e., the levels of disturbance appeared to increase markedly; thus, an altitude of no lower than 15 m.a.s.e. was adopted. Imagery captured at this flight altitude has a ground pixel resolution of 10 mm (i.e., each pixel on the screen represents 10 mm on the ground) and was deemed sufficient for this study (Figure 2 demonstrates the image quality achieved).

Following launch as described above, the protocol developed for each UAV flight was as follows:

1. A smooth flyover at 40 m above the subcolony with a take‐off site approximately 20 m to the side of the subcolony, allowing the birds to become comfortable with the UAV.
2. The altitude of the UAV was lowered to 15 m whilst in motion to the side of the subcolony.
3. A transect was flown at a speed of 3–4 ms^−1^ providing image overlap of approximately 20% over the subcolony with images captured at 2‐s intervals to ensure a similar overlap between images.

Subcolonies were flown either individually, or where practical, 2–3 of the smaller neighboring subcolonies were grouped and flown together.

### 2.4 Behavioral responses of gulls to UAV survey

Video recordings were taken of 12 individual subcolonies (each flown independently to avoid pseudoreplication of the analysis) before and during UAV surveys to quantify the impact of the UAV on the behavior of the gulls that were surveyed. It was decided that behavior resulting in birds leaving the ground would be most indicative of stress, rather than socializing and pair‐bonding behavior, and so analysis did not include ground‐based behavioral responses. These were analyzed into the following three categories of behavior to assess the effect of UAV flights:

1. Hop—a gull flies briefly (<10 s in flight), and low, to land elsewhere within the colony.
2. Flight—a bird takes flight from the ground in the colony and remains in the air for more than 10 s, typically remaining aloft, but not approaching the UAV directly.
3. Attack—a gull flies aggressively and directly toward the UAV. A single attack was noted during survey flights, lasting just 5‐s, and thus was not considered in analyses.

Counts of each behaviour were standardized as counts per minute of observation, during the period prior to the UAV flight (mean 1.084 ± 0.0093 min) and for the duration of the UAV survey (mean 5.26 ± 1.047 min). The differences between both flights and hops per minute before and during UAV surveys were not normally distributed (Shapiro–Wilk tests: flights *W* = 0.726, *p* = 0.0015; hops *W* = 0.785, *p* = 0.0063), and thus, Wilcoxon signed rank tests were used to compare behavior. A sample video of a traditional walk‐through count was also collected and used for comparison, although no statistical testing was undertaken on this.

### 2.5 Image processing

Photogrammetric data processing was carried out using the software Agisoft PhotoScan v1.2.4 to orthorectify the images and produce an orthomosaic (i.e., a mosaic image with positional accuracy) of each subcolony. Photoscan is a commercially available program that uses algorithms to automatically detect features in the images such as edges and points from the unordered aerial image collection (Siebert & Teizer, 2014); combining this with ground control data produces accurate digital surface models (DSMs; Fonstad, Dietrich, Courville, Jensen, & Carbonneau, 2013). Based on this model, it is able to convert the images into a single 2D orthomosaic without the individual scale, tilt, and relief distortions of each image.

All images were manually assessed prior to processing and, where necessary, deleted from the subset if they were distorted or blurred from the flying motion. All remaining images for each subcolony were added to the software, and image processing followed the recommended procedure outlined by Agisoft (2016). Alignment was carried out using the “high” setting to achieve the best possible accuracy, and “Generic” pair preselection was used to reduce processing time. If this failed, the “Reference” pair preselection parameter, in which the overlapping pairs of photographs are selected based on the measured camera locations, was used (Agisoft, 2016). Where automated alignment failed, chunks (subsets of the images) were created and aligned. Subsequent merging of these chunks was effective in most instances and thus permitted further processing. If the processing failed again, due to insufficient overlap between images, the unaligned images within the model were discarded, or where they were critical for the model, the entire model had to be rejected. Of the subcolonies flown, successful models were created for 14 of these, with eight subcolonies deemed inadequate for further processing, highlighting the importance of adequate overlap and sufficient transects during image capture.

**Figure 3:**
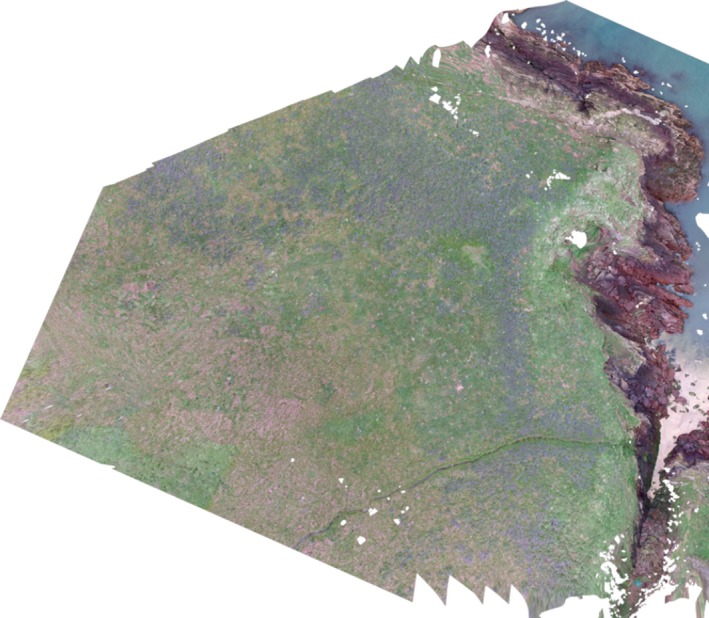
Example of an orthomosaic showing the North Haven subcolony (Colony 3)

A sparse point cloud was produced and georeferenced in the projection WGS 1984 using the camera's internal GPS and supplemented with manually collected GCPs that could be identified on the imagery; however as these were located on the edges of colonies (to avoid disturbance during collection), their impact was negligible. A dense point cloud was then produced using the “high” setting to maximize the geometric accuracy. Aggressive depth filtering was used to remove outliers from the dense point cloud, which is more efficient at retaining the birds particularly in vegetation. A 3D mesh with 10,000 faces was produced to reduce processing and complexity of the model whilst retaining sufficient detail. A DSM was produced, and then, an orthorectified aerial image was produced with color correction enabled based on this DSM (Figure 3). The orthomosaic was exported into ArcGIS v10.3.1 and reprojected into British National Grid.

### 2.6 Classification

Following the development of the 14 orthomosaics, a semiautomated classification was undertaken to count the number of *Larus fuscus* using supervised classification. This is a user‐driven process that involves acquiring a sample of pixels from a known class (known as a training set) from the image that provides an accurate representation of the class (e.g., the heads of the gulls; Foody & Mathur, 2006) to create a unique spectral signature for each class; the classification process then automatically separates the image into these. The *training sample manager* tool was used to identify the spectral signature of areas of interest within ArcGIS v10.3.1. Polygons were selected that represented different areas of interest (the backs, heads, and tails of the gulls) and combined to create a spectral signature for each. The maximum likelihood tool was used to perform the supervised classification following similar studies by Fretwell et al. (2012, 2014, 2017) and Grenzdörffer (2013). An iterative process of adding further spectral signatures to other surrounding features (such as bluebells, grass, and rocks) was followed. The statistics and histograms of each of the signatures’ color band were analyzed. Where a significant overlap existed, the histograms were narrowed and retested. The “reject fraction” parameter was varied and likewise retested to reject a portion of cells due to the lowest possibility of correct assignments allowing for straightforward removal of any unrequired pixels.

Following a maximum likelihood classification of the spectral signatures of the following classes, (a) the back portion of the gull; (b) the gulls’ head and tail; (c) bluebells; and (d) rocks and sea campion, with a reject fraction of 0.01, the gulls were selected and the majority of unwanted pixels excluded. Each additional orthomosaic was subsequently classified using the original signature set (C1) along with a new signature set from the specific orthomosaic under evaluation to test for effectiveness (C2). The classification output was converted into polygons to enable further analysis of the discrete classes that had been identified, and parameters were tested in ArcGIS and either discarded or retained to increase the accuracy of identification with the functions shown in Table 1, buffering was found to be the most important function. The model builder in ArcGIS was utilized to batch process all of the orthomosaics reducing user time greatly.

**Table 1:** The processes used in ArcGIS to improve the accuracy of gull extraction form within the orthomosaics and their perceived impacts

Processing function	Parameter defining the part to be erased	Positive impact on the file	Negative impact on the file
Removal based on the size of initial polygons	<0.0008 m^2^	Able to reduce a lot of background noise whilst retaining gulls features.	Removes small outlying areas of the gulls
Removal based on the distance from other class of gull from the other (i.e., head from back)	>3 cm	Removed much of the background, particularly bluebells that are not nearby polygons mistaken for the whiteheads.	Can remove outlying areas of the gulls.
Removal based on the size of merged polygons from head and backs	<0.0125 and >0.001	Removes areas of rock.	Fails if other areas fit within the bounding size. Has the potential to remove polygons of birds. The parameters could be widened but would result in extra manual cleaning up.
Buffering	1 cm	Allows merging of nearby polygons and reduces the likelihood of gulls being counted twice.	

**Figure 4:**
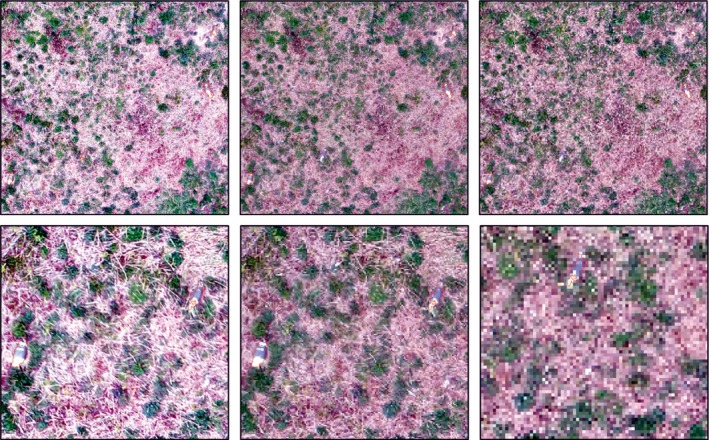
Automated identification of the same orthomosaic at three different grid cell sizes: 10 mm (left), 20 mm (center), 40 mm (right). The images show birds that are both identified and the greater proportion that are missed at 20 and 40 mm resolution

A shapefile with outlines of the objects identified as birds using both the C1 and C2 signature sets was overlaid over the original image to enable a short process of manual editing to be applied. The image was systematically scanned, and identified objects were checked to confirm that it was indeed a bird. If the object was not identified as a bird, the polygon was deleted. This process was relatively straightforward and fast to complete; the resulting layers are referred to as C1_e_ and C2_e_ for each subcolony. The counts for each method were retained both before and after a phase of manual editing. Orthomosaics were resampled to give cell sizes of 1, 2, and 4 mm to assess the best cell size for autoidentification. A cell size of two undercounted the birds by 3.5%, whilst 4 mm resulted in a 4.5% loss of birds (Figure 4).

**Figure 5:**
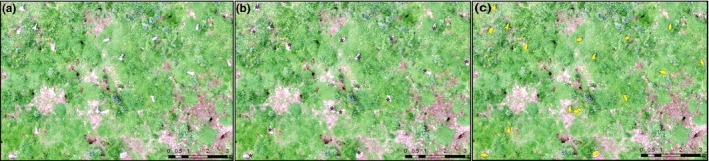
An example of the classification output accuracy: (a) the original orthomosaic, (b) the manual counts with the pink stars highlighting gulls, and (c) the classification results outlining the gulls in yellow

Each of the orthomosaics was counted manually using the method of Hodgson et al. (2016, 2018) to identify the number of gulls in each. The success of the classification was determined by comparison between these manual counts and the semiautomated classification outputs for both C1 and C2, as well as the further manual editing stage completed for each of these (see Figure 5). The orthomosaics were clipped to fit the subcolony extents in ArcGIS, digitized from a drawing provided by the reserve warden. However, it was not possible to compare the classification counts directly with the field point counts and walk‐through surveys as the subcolony boundaries were not exact (due to both the hand‐drawn maps used and the lack of image overlap at the edges of the colonies during image capture that resulted in not all colonies being re‐created in their entirety), the primary focus of the field surveys was on counts of nests rather than birds, and the field and UAV surveys were not undertaken at the same time so there would be some discrepancy with the birds recorded. This meant that a full statistical comparison between the field and computer bird counts for all of the subcolonies monitored was not possible.

Additionally, to address the third research question and explore the utility of this technique to identify different gull species, polygons were drawn to create a signature set of the three different gull species using a collection of the raw images. The same technique as previously described was used to classify the gulls and surrounding features, but the back portion of the gull was used to differentiate between the three species of *Laridae* present (*Larus argentus*,* L. fuscus,* and *Larus marinus*).

### 2.7 Statistical analysis

Variables were tested for normality (Shapiro–Wilk test) prior to analysis, and nonparametric tests were undertaken when variables failed (*p* < 0.05). Nonparametric correlations (Spearman's rank) between the semiautomated classification outputs and manual image counts were examined. Statistical analyses were carried out using IBM SPSS Statistics for Windows v24 and R v3.4.2.

## 3 RESULTS

### 3.1 Behavioral responses of gulls to UAV survey

**Figure 6:**
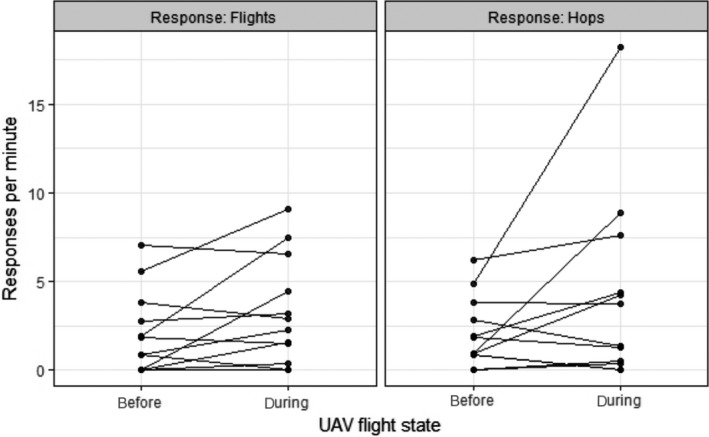
Behavioral responses of Lesser Black‐backed Gull before and during 12 unmanned aerial vehicle survey flights over subcolonies. There was no significant difference in the numbers of flights (before: 2.063.284 ± 3.024 per minute, during: 3.284 ± 3.024) or hops (before: 2.089 ± 1.964, during: 4.217 ± 5.303 per minute)

During observations of 12 gull subcolonies, no instances of nest loss due to predation or cannibalism were recorded, either before or during any of our UAV surveys, and there was only one short‐lived attack of a UAV made by a gull. We found no impact of UAV survey flights at 15 m on gull behavior: There was no significant difference in either the number of flights by gulls (Wilcoxon signed rank test: *V* = 17, *p* = 0.1682) or the number of hops within the colony (*V* = 24, *p* = 0.2661) between the period immediately prior to or during the UAV survey flights (Figure 6). By comparison, walk‐through counts caused all gulls in the subcolony to take flight at some point during the short (5–10 min) duration of the walk‐through, with many birds alarm calling and a few gulls attacking fieldworkers with a swooping flight and a peck to the head and/or defecation (pers. obs. by authors). We cannot comment on the physiological responses of the birds, and their behavior indicated that the birds were not visibly stressed during or after the UAV flights. Little general disturbance was noted by the authors and wardens to other bird species in the vicinity of the subcolonies; however, both Oystercatchers, *Haematopus ostralegus,* and Ravens, *Corvus corax,* did approach the UAV whilst alarm calling when they first encountered it, but this response lessened after the initial flight.

### 3.2 Semiautomated gull counts

**Figure 7:**
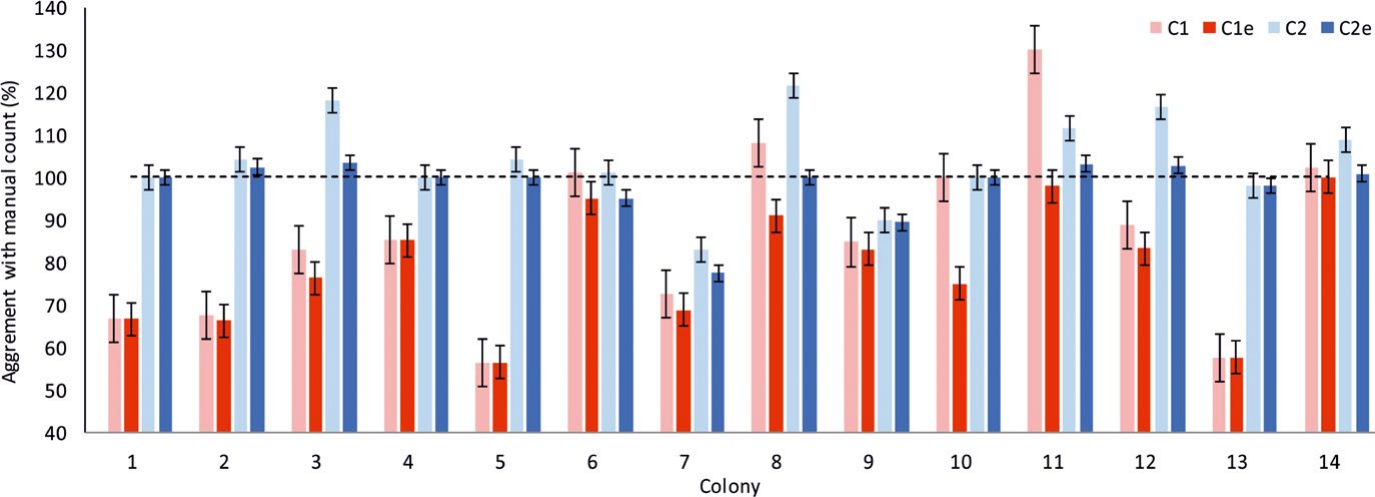
Variation between subcolonies in the agreement of semiautomated counting methods with manual counts. Overall correlation: C1 = 93% (*N* = 13, *r*_s_ = 0.934, *p* < 0.001), C1_e_ = 97% (*N* = 13, *r*_s_ = 0.968, *p* < 0.001), C2 = 96% (*N* = 14, *r*_s_ = 0.961, *p* < 0.001), C2_e_ = 99% (*N* = 14, *r*_s_ = 0.991, *p* < 0.001)

For the 14 orthomosaics created, the totals of the manual counts of images (“manual”) extracted from each subcolony classified are compared with the counts obtained through the semiautomated processing (C1 and C2) and additional manual editing that was applied to these (C1_e_ and C2_e_). Hodgson et al. (2016) found that manual counting of birds within images is consistently similar to or significantly larger than ground counts because of the downward‐facing perspective, although it should be noted this is likely to be species‐ and habitat‐specific. Thus, the correlation here between semiautomated and manual counts suggests that these should correlate and offer a real alternative for replacing time‐consuming point counts and providing counts of inaccessible areas (Figures 7).

Method C1 underestimates the number of birds in 10 of 13 subcolonies (76%), whilst C2 generally overestimates, with 11 of the 14 subcolonies having a count that was greater than the manual count (Figure 7). Counts from both methods were subsequently reduced following manual editing (C1_e_ and C2_e_). The mean agreement when a single signature set was applied was 86% (C1), compared to 104% (C2) from a signature developed from each image. These fell to 79% and 98%, respectively, once manual editing was applied to the results. The manual editing confirmed all objects to have been correctly identified as birds in four of the subcolonies in both methods, with the C1 set having a mean error of 7% incorrect identifications and C2 having a mean error of 5%. There was a significant positive correlation between semiautomated counts and manual eye counts in all cases (Figure 7).

**Figure 8:**
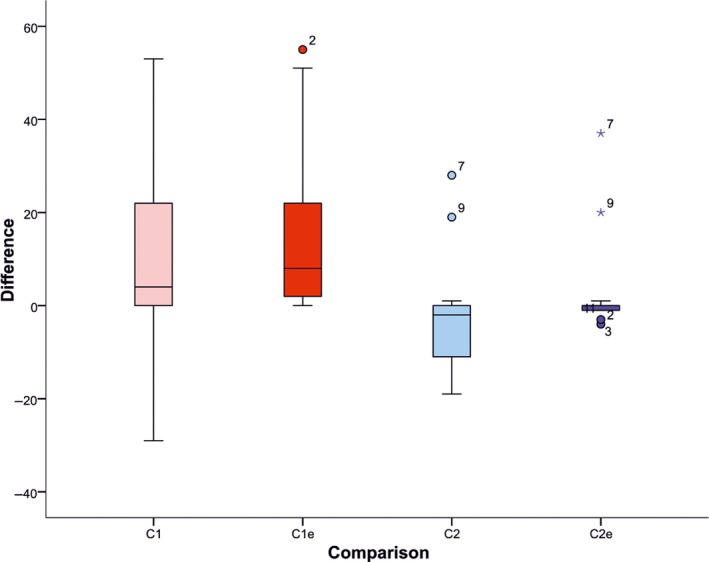
Differences between the manual counts and the semiautomated methods to count Lesser Black‐backed Gulls. Boxes represent the first and third quartiles, and the median is marked and labeled, and bars represent 1.5 times the interquartile range. Extremes are shown as circles and stars as outliers

The C2 method was the most proficient, bringing the lowest discrepancy between semiautomated counts and manual image counts, and the lower variance than method C1 (Figure 8). There was little variation in the counts between the subcolonies with the different background vegetation (shown in Figure 2), thus highlighting that the semiautomated classification technique was successful at detecting birds over a range of complex backgrounds. Variation between subcolony accuracy was primarily due to the image quality of the orthophoto.

### 3.3 Identifying gull species

**Figure 9:**
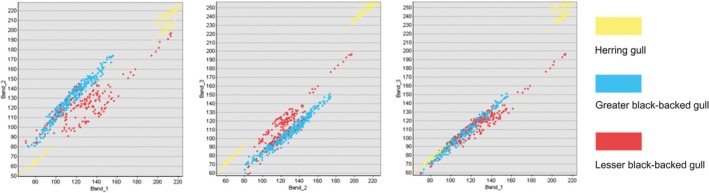
Scatterplots of the different color bands against each other of the three gull species (Band 1 = red, Band 2 = green, and Band 3 = blue), highlighting that there are clear differences between the species especially between Band 1–Band 2 and Band 2–Band 3

**Figure 10:**
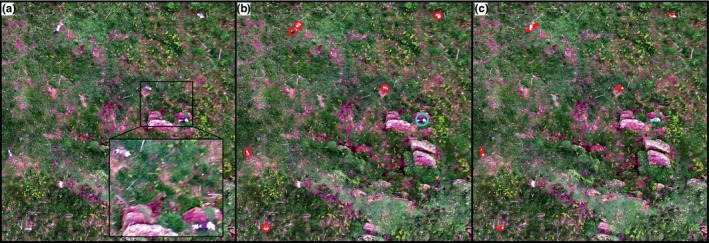
An example of the species classification output highlighting the Lesser (red) and Great (blue) Black‐backed Gulls: (a) the original orthomosaic, (b) the manual species identification, and (c) the semiautomated classification output

Two example subcolonies were selected to investigate the potential of the semiautomated classification process to differentiate between the three species of *Laridae*: Herring Gull *L. argentus*, Lesser Black‐backed Gull *L. fuscus,* and Great Black‐backed Gull *L. marinus*. The original purpose of the UAV survey was focused on counting Lesser Black‐backed Gulls, so although our imagery did not permit full statistical analysis of species differentiation of a mixed‐species colony, there was sufficient imagery containing multiple gull species to enable a proof‐of‐concept analysis. The images collected are all true color and therefore contain three bands (red, green, and blue); using the band combinations, it is possible to determine whether the classification will be able to clearly distinguish between the signatures of the three species. Figure 9 shows that there are clearly identifiable differences between the color bands of the three species, therefore indicating that this method is suitable for differentiating and counting the number of birds of each species, with Herring Gulls being particularly distinct from the other two gull species. The semiautomated classification process successfully identified the three species of gulls on the subcolonies analyzed, an example of this is shown in Figure 10 where we differentiate Lesser and Great Black‐backed Gulls, and therefore shows the potential that this method has for species identification in future investigations.

## 4 DISCUSSION

This paper demonstrates that UAVs can provide accurate counts (comparing manual vs. semiautomated bird counts using the UAV imagery) of a colony‐nesting Lesser Black‐backed Gulls, *L. fuscus* without undue disturbance, using semiautomated image processing. We also indicate a method to distinguish between *three Laridae* species nesting in the same location. Behavioral analysis quantified the impact of the UAV on *L. fuscus* breeding colonies, which was found to be very low to negligible when flying at or above 15 m over the subcolony. This noninvasive method has the ability to remotely record seabird species and enhance the survey toolkit that is already employed, with the orthorectified images providing a permanent record of bird location and number, and the classification process outlined enabling semiautomated counts of individual birds and species. There is potential to expand this technique to monitor other seabird species, with appropriate behavioral assessment, and to monitor colonies that are currently inaccessible for traditional point and walk‐through surveys at a relatively low cost, thus expanding the scope of the seabird monitoring program. UAVs could also prove useful in monitoring urban gull populations, which is a posing a challenge (Coulson & Coulson, 2015), but this would need to be carefully managed and potentially restricted to areas with low populations due to the restrictions around flying in built‐up areas. The methods developed and applied here offer a promising avenue to improve and expand avian census techniques, and further developments in technology and image processing techniques will no doubt increase their utility further.

Flying a UAV in a controlled manner in the presence of the experienced ornithologists has allowed a suitable method for bird surveys over breeding gull colonies to be developed. Vas et al. (2015) suggested that flying within 4 m of birds is feasible in certain situations. However, we found that flying below 15 m provided no useful increase in image quality that warranted the increased processing time that accompanied the increased photographs that were captured, and brief trials below 10 m caused undue disturbance to nesting gulls. An altitude of 15 m is therefore recommended for flying over breeding gull colonies with minimum disturbance to obtain images with a cell size of 10 mm. With best practice including a take‐off and landing site for the UAV away from the colony, and an initial acclimatization flight at 40 m before gradually lowering this to 15 m for the image collection, making sure to avoid any sudden drops in altitude that was perceived as predator behavior by the birds (Brisson‐Curadeau et al., 2017). Our UAV protocol has been successfully adopted to survey breeding Lesser Black‐backed Gulls using a fixed‐wing UAV with similarly minimal disturbance (A. Kilcoyne, Natural England, pers. comm.). With a higher resolution camera, it would be possible to fly at a greater altitude and obtain similar resolution. With technological developments improving the quality and size of cameras, it may be possible to capture similar resolution imagery from higher m.a.s.e. in future. The result would be minimized disruption to birds, less flight time, and less processing to produce the orthomosaics. Our image capture in this study was subject to edge effects; therefore, we recommend the flying of overlapping and perpendicular transects with suitable overlap on a preprogrammed flight route and with the flight path exceeding the colony boundary to avoid distortion at the edges having an impact on counts. Furthermore, GPS ground control points should be distributed around the colony and captured in at least three images.

Using a UAV for ecological surveys provides a method for efficient data capture, and as the images are recorded permanently, they can be referred back to in future years, to compare not only the number but also the position of nesting birds. The relatively low cost of UAVs (approximately £2k for a quadcopter in 2018) offers the potential to capture seabird colonies at a time of year and spatial extent of the researchers choosing. The UAV proved to be noninvasive and resulted in minimal disturbance to the birds within the subcolonies, and the overhead view provided by the images is ideally suited to observe birds in a range of habitats, including overgrown vegetation in which nests/birds can be difficult to spot from ground level. However, the importance of using both an experienced UAV pilot and a trained ornithologist to undertake bird surveys, at least in the first instance, is stressed and if this technique was to be used for other bird species, this would require further behavioral assessment and perhaps a modification to the minimum flying height used, as also recommended by Vas et al. (2015) and Brisson‐Curadeau et al. (2017).

**Figure 11:**
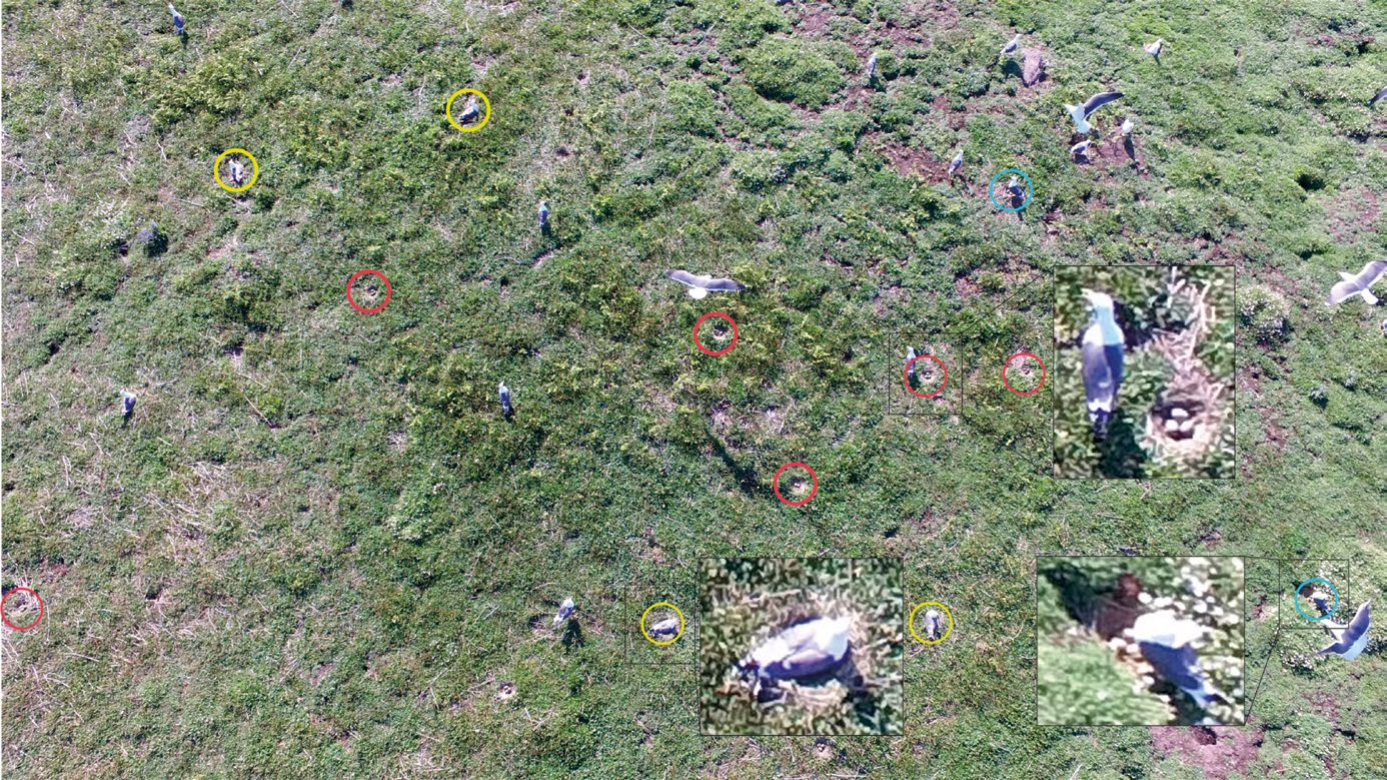
An example of an image captured at 5 m.a.s.e.: Red circles = nests that were temporarily abandoned with eggs clearly visible, blue circles = nests temporarily abandoned with nests and eggs partially visible, yellow circles = nests with apparently incubating adults. Insets show examples of close‐ups of individual birds

Unmanned aerial vehicle survey flights at 15 m had no significant effect on flight behavior in the colony as compared to ground observers being present within 50 m, which is regularly experienced by these birds being approached by island visitors on nearby paths. By comparison, walk‐through counts of gull nests typically result in a high level of disturbance and “attack” behavior by the gulls directed at fieldworkers (pers. obs. by authors), although there are no current alternative methods for estimating productivity. Figure 11 shows the potential utility of the images at 5 m whilst also demonstrating the impact on the birds; the majority of incubating birds have left the nest, indicating high stress although they remained in attendance and returned to nests as soon as the UAV was no longer directly overhead. However, this provided the opportunity to survey the number of nests and eggs, potentially useful information in survey work and understanding in population studies, and potentially separating breeders from nonbreeders and partner birds both on and off the nest, as has been demonstrated in Common Murres, *Uria aalge* (Brisson‐Curadeau et al., 2017). However, this presents a risk of a collision between the UAV and birds that does not exist in a walk‐through count. The complex costs and benefits of flight plans in UAV survey will vary between types of equipment, species, and sites, necessitating careful consideration and ideally trials of the impacts of UAV survey on wildlife.

The semiautomated image classification method presented here has the ability to identify and count the number of individual birds within gull colonies, as well as offering the potential to differentiate gull species. Manual counting of birds in an image, similar to that employed elsewhere by Hodgson et al. (2016, 2018), Sardà‐Palomera et al. (2012), and Chabot et al. (2015), proved to be time‐consuming in our study system and more prone to errors. For example, semiautomated classification identified birds that were not picked up by manual counting in four subcolonies.

Using Agisoft PhotoScan produced 14 useable orthomosaics and demonstrated that the method and software can produce suitable orthomosaics for further processing if adequate imagery is collected. Importantly, creating an individual training set for each orthomosaic, similar to the method promoted by Fretwell et al. (2012, 2014, 2017), improves the ability to recognize individuals. In an ideal scenario, all images would be collected under the same environmental conditions allowing for a single training set to be used; however, this is unrealistic in practice; different light conditions, cloud cover, and shadows create differences in the color bands that are hence best recognized by creating a signature set based on those exact conditions. Of the 64 individuals missed by the semiautomated counts (of a total 1,183) using this classification method, all were located in three subcolonies, two of which contained large distortion within the images, suggesting an excellent level of accuracy from this method and emphasizing the importance of the collection of well‐distributed, high‐quality images.

The subsequent procedure of applying a set of parameters was successful in removing much of the noise during the classification process whilst retaining the bird identification, despite some overlap between the spectral signatures of the birds with the background habitat, particularly the bluebells. The parameters that were set here retain only a small amount of noise without missing individuals. Clearly, if the method is to be applied to other species then alterations to the parameters used and potentially new parameters will have to be explored and applied. A final manual stage to remove the remaining noise is recommended to increase the accuracy of the final output; this procedure was relatively quick and easy; it was clear in virtually all cases which objects were not birds based on their shape, position within the frame, or position related to other objects. This process of manual editing proved important in this research in improving the accuracy and precision to a mean agreement of 98% and a correlation of 99% with manual counts.

Preliminary investigation of the functionality of identifying different gull species suggests that the spectral signature of each gull species is sufficiently different such that it is possible to create a training set that can identify individuals of Herring Gull, Lesser Black‐backed Gull, and Great Black‐backed Gull. Indeed, given the advantage of overhead imagery, the potential for similar surveying of other species of nesting birds in similar or other habitats could be explored. Best practice will have to be established in terms of flying over other species and should be practiced in a similar way as here with experienced ornithologists being part of the team monitoring the bird behavior.

## CONFLICT OF INTEREST

None declared.

## AUTHOR CONTRIBUTIONS

GR, LC, and MW conceived the study and designed methodology; GR and LC collected and analyzed the UAV data and performed the semiautomated classification; MW collected the behavioral data; MS analyzed the behavioral data; and GR led the writing of the manuscript. All authors contributed critically to the drafts and gave final approval for publication.

## ARCHIVING DATA

The semiautomated classification model and associated data will be archived on Figshare (www.figshare.com) and the institutional repositories at the University of Gloucestershire and the University of York.
